# The Significance of Vascular Pathogenesis in the Examination of Corticobasal Syndrome

**DOI:** 10.3389/fnagi.2021.668614

**Published:** 2021-05-04

**Authors:** Anna Dunalska, Julia Pikul, Katarzyna Schok, Katarzyna Anna Wiejak, Piotr Alster

**Affiliations:** ^1^Department of Neurology, Medical University of Warsaw, Warsaw, Poland; ^2^Students’ Scientific Association of the Department of Neurology, Medical University of Warsaw, Warsaw, Poland

**Keywords:** tauopathies, corticobasal syndrome, corticobasal degeneration, vascular, neurodegeneration

## Abstract

Corticobasal syndrome (CBS) is a clinical entity, classified as an atypical Parkinsonism, characterized by both motor and higher cortical dysfunctions. The clinical manifestation of CBS is associated with several pathologies, among which corticobasal degeneration (CBD) is the most common. The aim of our study was to elaborate on the possible vascular pathogenesis of CBS and consider types of vascular lesions in these cases. Several cases of vascular CBS are described in the literature. The majority of presented patients were affected by internal carotid artery (ICA) stenosis and ischemic strokes; few cases were associated with vascular malformations or autoimmune diseases. Vascular CBS is preceded by an abrupt onset. The clinical manifestation does not significantly differ with non-vascular CBS. Patients with vascular CBS are usually elderly; often with coexistent hypertension, dyslipidemia and diabetes mellitus. Inferring from our observations, cerebral hypoperfusion can play a significant role in neuropathological changes in neurodegenerative diseases. To the best of our knowledge paper is the first comprehensive review of vascular CBS and we are positive that our observations show that further research concerning the vascular pathogenesis of tauopathy atypical Parkinsonism is required.

## Introduction

Corticobasal syndrome (CBS) is a clinical entity, classified as an atypical Parkinsonism. The research in the topic of CBS is relatively recent. It was primarily described as corticobasal degeneration (CBD), as Rebeiz et al. (1967, 1968) presented studies about then so called “corticodentatonigral degeneration with neuronal achromasia.” Neurodegeneration was, thereafter, incorporated in the nomenclature of this phenomenon and used to describe the etiology of the symptoms, which are nowadays comprised in a clinical entity – CBS (Gibb et al., 1989). CBS is characterized by both motor and higher-cortical dysfunctions. Armstrong et al. (2013) presented the criteria for the diagnosis of CBS, which indicate two main domains of this syndrome: cortical dysfunction with orobuccal/limb apraxia, cortical sensory deficit, alien limb phenomena and extrapyramidal dysfunction expressed as: limb rigidity or akinesia, limb dystonia, limb myoclonia. The diagnosis of probable CBS can be made if two of cortical dysfunction features and two of extrapyramidal dysfunction features, with asymmetric distribution, are present, whereas possible CBS is characterized by one of cortical dysfunction features and one of extrapyramidal dysfunction features, which can affect the body symmetrically. Abovementioned criteria partly correspond with another diagnostic tool (Mathew et al., 2011) known as Cambridge criteria, in which akinetic rigid syndrome, limb apraxia and speech/language impairment serve as main symptoms and are accompanied by minor ones: focal or segmental myoclonus, symmetrical dystonia, alien limb phenomenon, cortical sensory loss or dyscalculia, frontal executive dysfunction and visuospatial deficits.

Epidemiological data concerning CBS is limited, but based on conducted studies its incidence is estimated to be less than 1 per 1,00,000 patients per year. Commonly the age of onset of CBS is between 50 and 70. The only known risk factor is advanced age (Grijalvo-Perez and Litvan, 2014).

CBS is associated with CBD, but these terms must be distinguished, as the first one is a clinical diagnosis, while the second one is a neuropathological term (Saranza et al., 2019). CBD describes a pathology in which abnormally hyperphosphorylated 4R tau protein is deposited in the somatosensory cortex, premotor, supplementary motor cortices, brainstem and basal ganglia (Ali and Josephs, 2018). The clinical manifestation of this disease depends on the localization of neuronal loss and tau pathologies in the brain (Saranza et al., 2019). Prevalent clinical phenotype of CBD is CBS; however, CBD may also manifest with progressive supranuclear palsy syndrome (PSP), frontotemporal dementia (FTD), Alzheimer’s like dementia (AD), the frontal behavioral-spatial syndrome and the non-fluent/agrammatic variant of primary progressive aphasia (Armstrong et al., 2013; Ali and Josephs, 2018). On the other hand, CBS can also be related to the spectrum of other neuropathologies such as AD, PSP, Pick’s disease (PiD), fronto-temporal lobar degeneration with ubiquitin–TDP-43-positive inclusions (FTLD-TDP), Lewy body disease (LBD), and Creutzfeldt–Jakob disease (CJD) and globular glial tauopathy (Boeve, 2011; Parmera et al., 2016; Marsili et al., 2018).

Because of the complexity of the abovementioned phenomena and their mutual superimposition, it should be mentioned that PSP and CBD have overlapping phenotypes and therefore are often difficult to differentiate. The new approach of “probable 4-repeat (4R)-tauopathy” nomenclature has been introduced for joint clinical diagnosis of PSP and CBD by Respondek et al., who reached high specificity of this new diagnostic category in their study. Both, CBD and PSP, are 4R tauopathies, however, the localization of neurodegeneration and deposits of tau pathologies in them vary, especially in the case of astrocytic tauopathy deposits (Respondek et al., 2020). In CBD the most characteristic glial lesion are astrocytic plaques, whereas in the PSP more characteristic is the tufted astrocyte. Astrocytic plaques are seen especially in the cortex, but also in the caudate and putamen. In PSP tufted astrocytes mostly accumulate in precentral gyrus, striatum and superior colliculus. Another neuropathological feature which differentiates PSP and CBD is presence of ballooned neurons in CBD, whereas in PSP they are rare. In PSP more common than in CBD is presence of coiled bodies, which are oligodendroglial tau inclusions (Irwin, 2016; Parmera et al., 2016). Many of genetic risk factors and pathological mechanisms are common for CBD and PSP. Clinically, they can present like Richardson’s syndrome, CBS, frontal cognitive/behavioral presentation, and speech/language dysfunction (Respondek et al., 2020). In comparison to 4R tauopathies, PiD is characterized by the presence of three-fold (3R) tau isoforms, while in Alzheimer’s disease both 3R and 4R isoforms are found in the filaments (Goedert et al., 2017). Thereupon, it can be concluded, that CBS can be a manifestation of 4R and 3R tauopathies.

Among the typical features of CBS there is also an asymmetric distribution of the motor symptoms. According to Di Stasio et al. (2019) this kind of typical pattern corresponds with decreased glucose metabolism and cerebral blood flow in positron emission tomography (PET) and single photon emission computed tomography (SPECT), as the affected side of frontoparietal cortex, caudate, putamen, thalamus, pons, and cerebellum is often contralateral to the clinically more symptomatic side (Abe et al., 2016).

## Materials and Methods

A comprehensive literature search for articles was performed using the PubMed database. The following keywords were used: “CBS,” “CBD,” CBS diagnostic criteria,” “vascular CBS,” “vascular progressive supranuclear palsy,” “vascular tauopathies,” “vascular Alzheimer’s disease,” “vascular Parkinsonism,” “tauopathies,” “cerebrovascular frontotemporal lobar degeneration.” The search was limited to human subjects with language restriction to English and French from 1994 to December 31, 2020. The snowball strategy, including a manual search of the references listed by studies retrieved from the online databases and from previously published reviews, was also performed to identify potential additional studies. The following inclusion criteria were used: studies published in the English and French language, and studies available in full text.

## Results

### Vascular Pathogenesis of Tauopathies

Tauopathies are neurodegenerative diseases, which are morphologically, biochemically, and clinically heterogeneous, however, their common feature is the accumulation of abnormal tau protein in the brain (Tan et al., 2018). They comprise following disorders: AD, a 3R/4R tauopathy, and frontotemporal lobar degeneration subtype tau (FTLD-tau) group, which includes: PSP, CBD, argyrophilic grain disease (AGD), all considered 4R tauopathies, and PiD, a 3R tauopathy (Kovacs, 2015; Arendt et al., 2016; Irwin, 2016; Kovacs, 2017). Interestingly, Mulroy et al. (2020) described the broad spectrum of tauopathies, in which the accumulation of tau protein is secondary to the pathophysiology such as head trauma, ischemia, oxidative stress and alterations in protein synthesis. It should be emphasized that the number of tau-related disorders is constantly increasing. Numerous attempts have been made in order to assess the incidence and potential effect of vascular abnormalities in abovementioned diseases. Although some patients with FTLD had cerebrovascular changes, the neuropathological studies did not find the association between FTLD and cardiovascular disease (CVD) (Baborie et al., 2011; De Reuck et al., 2012). Vascular PSP can be identified in the presence of CVD, when cardinal pathologic features indicating idiopathic PSP cannot be found. Clinical symptoms of vascular PSP can differ from those presented in idiopathic PSP. Multiple cerebrovascular lesions were found in vascular PSP, especially in thalamus and basal ganglia, while the main areas affected in patients with idiopathic PSP are substantia nigra, subthalamic nuclei, and periaqueductal gray matter (Josephs et al., 2002). The most common risk factors in vascular PSP are hypertension and smoking (Rabadia et al., 2019). Moreover, male gender is predominant in patients with vascular PSP (Winikates and Jankovic, 1994) and it can indicate the role of male gender in CVD. In addition, hypertension can cause deterioration of tau-mediated motor impairment. However, vascular or white matter lesions are responsible for the exacerbation (Díaz-Ruiz et al., 2009). The vascular PSP called “Mokri syndrome” is also worth mentioning. It is a rare disorder appearing after ascending aorta surgery. The typical symptoms include supranuclear gaze palsy, gait imbalance and dysarthria (Mokri et al., 2004; Tisel et al., 2020). AD differs from other tauopathies in the presence of β-amyloid peptide, but not only the neurodegenerative basis of this disease but also the correlation between AD and CVD attracts interest of the researchers in this field (Liesz, 2019). According to de la Torre, chronic brain hypoperfusion, which can be caused by hypertension or carotid atherosclerosis, can initiate AD (De La Torre, 2018). Studies showed that reduced oxygen level leads to increased β-secretase activity and induces the formation of β-amyloid protein. Moreover, CVD can lead to local hypoxia or even ischemia, which induce expression of hypoxia-inducible factor-1α (HIF -1α). According to recent studies HIF-1α stimulates the expression of β-secretase (Khan and Davies, 2008; Salminen et al., 2017). Furthermore, several cardiovascular genes that increase the risk of AD have been identified by genetic studies (Broce et al., 2019). Interestingly, AD in comparison to FTLD has a higher prevalence of CVD (Toledo et al., 2013). As mentioned above, AD can present clinically with CBS. Clinical symptoms such as tremors, myoclonus and early memory impairment can suggest CBS-AD (Chand et al., 2006; Hu et al., 2009). Magnetic resonance imaging (MRI) findings showed atrophy not only of subcortical gray nuclei and basal ganglia, but also temporoparietal region that is characteristic for CBS-AD (Josephs et al., 2010). We found it worth mentioning because there are many studies on vascular pathogenesis of AD and that can suggest the association between CVD and CBS.

### Causes of Vascular CBS

#### Carotid Artery Occlusion

Carotid artery occlusion has been reported to be one of the potential causes of CBS. In the case report described by Engelen et al. neurological symptoms (Table 1) were associated with decreased left internal carotid artery (ICA) flow. Acute appearance of these symptoms suggest vascular, rather than neurodegenerative, etiology, as CBD tends to have insidious onset (Engelen et al., 2011). Marques et al. described the occurrence of unexplained hemiplegia with consecutive homolateral CBS in two patients with metabolic disorders and prior endarterectomy due to ICA stenosis. In spite of the absence of visible ischemic lesions in these patients, it can be hypothesized that iatrogenic transient hypoxia and consequent pathologic change in neuronal networks of cortex and basal ganglia resulted in CBS (Marques et al., 2018). Additionally, other authors Vitt et al. (2020); Miyaji et al. (2013), and Kim et al. (2009) described patients with symptoms suggesting CBS with ICA stenosis (Table 1). In the case observed by Kim et al. (2009), authors assumed that lesions in brain MRI were typical for progressive ischemia due to large vessel steno-occlusion, although the mechanism is unclear. Abovementioned cases, despite being relatively uncommon, suggest that the ICA occlusion and consecutive depletion of the cerebral blood flow in certain brain regions, can lead to the occurrence of CBS-like symptoms, ranging from motor abnormalities to higher-cortical dysfunctions. Blood flow reduction, due to stenotic ICA, was linked to pathologic state of increased oxygen extraction, called misery perfusion, in which maximal vasodilation enables the oxygen supply. It cannot be excluded that this kind of changes lead to selective dendritic and cortical neuronal injury, manifesting among others in CBS-like symptoms (Yamauchi et al., 2007; Yamauchi et al., 2017). Some patients, who developed symptoms of CBS, suffered from hypertension. Marques et al. (2018) suggested that dysregulation of the cerebrovascular system and hypertension together lead to an increase of cerebral blood flow, which could potentially cause brain injury and support vascular etiology of CBS.

**TABLE 1:** Characteristics of the patientswith vascular CBS.

Authors	Clinical manifestation	Imaging and additional tests	Pathological examination	Patients’ characteristics
Engelen et al. (2011)	– Right-sided limb-kinetic apraxia and extrapyramidal dysfunction	– Left frontoparietal atrophy with multiple subcortical hyperintensities, left ICA stenosis	– No data	– 64-year-old man, cigarette smoker with hypertension
Marques et al. (2018)	– Myoclonus, left-sided apraxia, akinetic-rigid syndrome, rapid cognitive decline – tremor of the left superior limb with rigidity and bradykinesia, verbal and visual memory impairment, visuoc-onstructive apraxia and left-sided hemineglect	– 80% right ICA stenosis, right carotid endarterectomy, decreased metabolism in the right frontoparietal area and right striatum – left ICA thrombosis, right carotid endarterectomy, decreased metabolism in the right frontoparietal area and right striatum	– No data – no data	– 67-year-old man, history of two episodes of transient amaurosis of the right eye, diabetes mellitus, hypertension and dyslipidemia – 71-year-old man, history of a stent for ischemic heart disease and a left middle cerebral artery stroke 7 years previously, diabetes mellitus, hypertension and dyslipidemia
Vitt et al. (2020)	– cognitive decline, gait abnormality, asymmetric Parkinsonism and alien limb phenomena	– right ICA stenosis, right MCA territory infarcts with surrounding encephalomalacia and atrophy	– No data	– 81-year-old man with history of diabetes, hypercholesterolemia and hypertension
Miyaji et al. (2013)	– Right-sided limb-kinetic apraxia, constructional apraxia, right upper limb rigidity and a wide-based gait with small steps, aphasia, dysarthria, and right hemiparesis	– Left ICA stenosis, decreased cerebral blood flow in the left hemisphere and crossed cerebellar diaschisis, ischemic lesions in the left corona radiata and the left watershed area	– No data	– 65-year-old man with diabetes mellitus
Kim et al. (2009)	– Left hand dystonia, generalized myoclonus, mild left side weakness, bilateral limb ataxia, dressing and ideomotor apraxia, rapid progressive memory loss and tendency to fall	– Diffuse cortical high signal intenities with intravascular enhancement at the frontal, parietal, occipital and temporal lobes, bilaterally, but predominantly in the right hemisphere, steno-occlusions of the right distal ICA and MCA and severe stenosis of the left MCA	– No data	– 75-year-old woman with hypertension, angina pectoris and bipolar disease
Koga et al. (2019)	– Spastic-dystonic left hemiparesis and hyperreflexia with clonus, left-sided apraxia, dystonic posture on the left extremities – cognitive impairment, marked asymmetric ideomotor apraxia in both upper extremities (left > right), rigidity (left > right), bradykinesia, myoclonus, possible eyelid apraxia or blepharospasm, neck rigidity, horizontal and up-gaze nystagmus, pseudobulbar affect, lower body negative myoclonus – asymmetric rigidity (right > left), lower muscle weakness (right > left), a Babinski sign on the right, slurring of speech, word-finding difficulties and short-term memory problems	– Asymmetric right hemisphere atrophy in the frontal, temporal, parietal and occipital lobes, right lateral ventricle dilation, hyperintensities of the periventricular region (gliosis), low signal changes in the right basal ganglia, right cerebral peduncle and right aspect of the pons – deep white matter hyperintensities consistent with small vessel disease, chronic lacunar infarcts, and mild cerebral atrophy – 90% left ICA stenosis, chronic infarct in the left frontal lobe, left frontal hypoactivity, diffuse left cerebral hypoactivity, including decreased activity in the left basal ganglia, thalamus and the right cerebellum	– No arteriosclerotic small vessel disease, Alzheimer-related pathology insignificant, tau, α-synuclein, and TDP-43 pathology not detected, infarcts in right hemisphere, Wallerian degeneration in right hemisphere – arteriosclerotic small vessel disease present, Alzheimer-related pathology insignificant, tau, α-synuclein, and TDP-43 pathology not detected, infarcts in left hemisphere, Wallerian degeneration in left hemisphere – arteriosclerotic small vessel disease present, Alzheimer-related pathology insignificant, tau, α-synuclein, and TDP-43 pathology not detected, infarcts in both hemispheres, Wallerian degeneration in left hemisphere	– 69-year-old man with homocystinuria, dyslipidemia, diabetes mellitus and migraine – 82-year-old woman with atrial fibrillation, hypertension, hyperlipidemia, diabetes mellitus, aortic atherosclerosis, congestive heart failure, coronary artery disease, peripheral vascular disease, chronic kidney failure and obstructive sleep apnea – 69-year-old man with hypertension, hyperlipidemia, stroke at age 51, severe peripheral and ischemic heart disease with two cardiac stents, peripheral cyanosis, claudication and colorectal cancer
Kreisler et al. (2007)	– Myoclonus and rigidity of the left upper limb, akinesia, loss of the swing of the left arm, apraxia and dysarthria, apraxia of the upper left limb, kinetic cerebral syndrome of the upper limbs – resting tremor, akinesia and rigidity of the left upper limb, gait instability, amimia, hypophonia, parkinsonian dysarthria, Parkinsonism, myoclonus, apraxia, dystonia, alien limb phenomena and cognitive decline – akinetic/rigid parkinsonian syndrome of the left side, gait abnormalities, myoclonus of the right upper extremity, apraxia and left hemiasomatognosia, right-left agnosia, dyscalculia – akinesia, rigidity, dystonia, tremor of the upper right limb, gait abnormalities, left apraxia, left spatial neglect, dyscalculia, dysexecutive syndrome – left Parkinsonism, mostly unilateral rigidity, akinesia, dystonia, left apraxia and dysexecutive syndrome	– Right hemisphere: ischemic lesions of frontal, pre- and post-central, temporal gyri, centrum semiovale, internal capsule; posterior fossa: ischemic lesions of cerebellar hemispheres and the left part of the mesencephalon – right hemisphere: ischemic lesions of the pre- and post-central gyri, centrum semiovale, putamen and caudate nucleus; left hemisphere: ischemic lesions in pre- and post-central gyri, parietal cortex, centrum semiovale, internal capsule, putamen and caudate nucleus; posterior: ischemic lesions of the pons – right hemisphere: ischemic lesions of frontal, pre- and post-central gyri, medial and lateral parts of the parietal lobe, insular cortex, occipital lobe, anterior, median and posterior parts of the centrum semiovale and putamen. left hemisphere: ischemic lesions of frontal, pre- and post-central gyri, the inferior parietal lobulus, insular cortex, anterior, median and posterior parts of the centrum semiovale, putamen, and thalamus – right hemisphere: ischemic lesions of frontal gyri, medial and lateral parts of the parietal lobe; and the median and posterior parts of the centrum semiovale. left hemisphere: ischemic lesions of the median and posterior parts of the centrum semiovale – right hemisphere: ischemic lesions of the anterior and median parts of the centrum semiovale, putamen, globus pallidus, caudate nucleus; left hemisphere: ischemic lesions of the anterior, median and posterior parts of the centrum semiovale, putamen, globus pallidus	– No data – no data – no data – no data – no data	– 64-year-old woman with mixed cryoglobulinemia type II, purpura of the lower limbs, and arthritis of the hip – 77-year-old women with hip replacement, breast neoplasia and hysterectomy – 76-year-old woman with hypertension – 68-year-old woman with mammary neoplasia and subocclusive fecal syndrome – 73-year-old woman with hypertension, hypercholesterolemia and transient global amnesia
Ling et al. (2010)	– Right hand apraxia and alien limb phenomena	– Infarction of the left middle cerebral artery territory in addition to the characteristic pathological changes associated with CBD	– Cerebral infarction of the left middle cerebral artery territory in addition to the characteristic pathological changes associated with CBD	– –
Morris and Lees (1999)	– Parkinsonism with hypomimic facies, shuffling gait, left-sided akinetic-rigid syndrome, vertical supranuclear gaze palsy, left-sided upper motor neuron facial weakness and asymmetric upper limb rigidity and bradykinesia (left arm more severely affected than the right one), left mild pyramidal weakness and apraxia	– Multiple infarcts in the cerebral hemispheres and basal ganglia with prominent lesions in the right parietal lobe and head of the left caudate nucleus	– No data	– 44-year-old woman with migraine
Martino et al. (2006)	– Ideomotor and limb-kinetic apraxia bilaterally, dyscalculia, difficulty in distinguishing left from right, walk with short steps, stooped posture, flexed dystonic posture of the right arm, retropulsion, hypomimia, intellectual deterioration	– Periventricular white matter changes, diffuse cerebral cortico-subcortical atrophy, several infarcts in both hemispheres, including striatum	– Non-specific severe gliosis of cortex, white matter and deep gray nuclei, without evidence of vasculitis, Immunohistochemical stains for amyloid and tangles (A4 and Tau) were negative	– 56-year-old woman with hypertension and migraine
Hashimoto et al. (2018)	– Asymmetric upper limb akinesia and rigidity more severe on the left side, dystonia of the left arm and leg, slow gait with absent arm swing, bilateral limb-kinetic apraxia prominent on the left side,	– Subcortical white matter ischemic changes, marked cortical atrophy that was prominent in the bilateral central areas, and several small infarcts in deep white matter of both hemispheres	– No data	– 53-year-old woman
Lee et al. (2014)	– Progressive clumsiness of the left hand, cognitive decline, numbness of the 4th and 5th finger on the left hand, dysarthria, memory loss and difficulty in calculation and mapping, oculomotor apraxia, dystonic posturing of both hands, moderate rigidity in only the left upper extremity, moderate bradykinesia bilaterally (more severe in the left upper and lower extremities), ideomotor and limb-kinetic apraxia bilaterally	– Widespread cortical atrophy that was more severe in the right fronto-parieto-occipital region without apparent changes in the periventricular white matter	– No data	– 47-year-old man
Bihari et al. (2016)	– Alexia, apraxia, agrammatism of the speech with mild anomia, executive dysfunction, hypokinesis with mild right upper limb rigidity, and postural instability and wide-based gait with decreased right upper limb synkinesis	– The hypointense subpial rim around the medial temporal structures and the pons, the right-sided thalamic cavernoma	– Aberrant vascular structures with hemosiderin deposition and gliosis	– 56-year-old woman with hypertension and migraine
Takase et al. (2017)	– Moderate cognitive decline, small voice, left dominant lead pipe rigido-spasticity, mild left hemiparesis, small steppage gait, micrographia, left limb-kinetic apraxia and constructional apraxia	– Multiple dural arteriovenous fistulas (DAVFs) around the superior sagittal sinus, dilatation of the superficial temporal arteries, bilateral hyperintense lesions in the subcortices, striata, thalami, and pyramidal tracts of the pons, microbleeds in the same regions, areas of severe hypoperfusion in the right fronto-parieto-temporal lobes, striatum, and thalamus	– No data	– 75-year-old man
Jain and Khan (2017)	– Spastic speech, asymmetric rigidity (L>R), myoclonus, dystonia with fixed twisting posture of left hand associated with alien limb phenomenon, bilateral cerebellar signs	– Asymmetrical hyperintensities in fronto-occipito-parietal region (cortical ribboning) with subtle hyperintensities also present in left temporal lobe – anti-TPO antibody titers raised to 590 IU/ml	– No data	– 70-years-old female
Sheetal et al. (2016)	– Difficulty in finding words, amnesia for recent events, inattention, difficulty in walking, psychomotor withdrawal, bradykinesia, agrammatical, effortful speech, four limb rigidity, more on the right side, focal myoclonus of the right upper and lower limbs during rest, aggravated by action and postures, severe postural instability, asymmetric Parkinsonism	– Anti-TPO antibody titers raised to 660 IU/ml	– No data	– 66-year-old female

*ICA – internal carotid artery, MCA – middle cerebral artery, TPO – thyroid peroxidase, TDP-43 – transactive response DNA binding protein 43 kDa.*

#### Ischemic Incidents

Evidence of cerebrovascular disease, in a form of ischemic areas, as a potential cause of CBS, have been provided by the research conducted by Koga et al. (2019). Postmortem examination of 217 patients with CBS diagnosis in 3 individuals displayed presence of infarcts in the brain regions (Table 1), potentially significant for CBS etiology. Another study involving postmortem examination was performed by Thal et al. (2015) and resulted in a finding that 33.3% of the patients with antemortem diagnosis of CBD had infarctions present in the brain, whereas no evidence of hemorrhage lesions was observed in this sample. Moreover, Kim et al. (2009) described vascular lesions of the ischemic character, located in fronto-temporo-parieto-occipital lobes in a patient with CBS. Ischemic pathogenesis of CBS was also mentioned in a case report by Peters et al., who described the occurrence of unilateral rigidity, micrographia and gait disturbances in a 56-year-old patient. Those symptoms, primarily ineffectively treated with levodopa/benserazide and selegiline, eventually emerged as caused by ischemic lesion located in the left cerebral peduncle, in close proximity to the substantia nigra and nucleus ruber (Peters et al., 2001). Further information about vascular etiology of CBS symptoms derives from the article by Kreisler et al. (2007). All of the patients had motor and higher-cortical dysfunction suggesting CBS and related ischemic lesions in the brain (Table 1). Another study, by Ling et al. (2010), assessed the possible pitfalls in CBD diagnosis and supplied with further information about potential vascular causes of CBS-like symptoms, as one patient’s right hand apraxia and alien limb phenomena eventually emerged as being partly attributable to infarction of the left middle cerebral artery territory. In conclusion, damage of the areas of the brain involved in praxis, such as frontal and parietal cortices, thalamus, basal ganglia, and white matter tracts between them, as well as degeneration of the corticospinal tract can present as CBS. It is possible, that lag between stroke and symptoms’ onset is present, as high-risk patients tend to experience silent ischemic incidents, which cumulatively deteriorate their neurological state. The presence of unexplained asymmetric spasticity and dystonia in the absence of cortical sensory loss, bradykinesia or cogwheel rigidity may be red flags for vascular CBS (Koga et al., 2019).

These clinical findings partially correspond with the research on functional SPECT imaging in CBS and related disorders, in which asymmetrical blood flow to the cerebral cortex, especially frontoparietal lobes, and basal ganglia in CBD was observed, in opposition to PSP, in which decreased blood flow was more of bilateral distribution (Zhang et al., 2001; Hossain et al., 2003; Ogawa et al., 2018). Okuda et al. (2000) share similar data, as more extensive and asymmetric blood flow reductions were observed by them in CBD than in PSP patients, with distribution in inferior prefrontal, sensorimotor, and posterior parietal cortices, but not in the subcortical regions.

#### Autoimmune and Other Rare Backgrounds

Additionally, vascular etiology of CBS may be attributable to antiphospholipid syndrome (APS) – autoimmune disease, which increases the general risk of thrombosis, also in the cerebral vessels (Carecchio et al., 2014). Despite the limited evidence of CBS in patients with APS (Ricarte et al., 2018), Morris et al. described a 44-year-old woman with Parkinsonism with hypomimia, gait disturbances, apraxia and a left-sided akinetic-rigid syndrome. Diagnostic investigation revealed, among others: the presence of lupus anticoagulant, elevated anticardiolipin antibodies and the presence of antinuclear antibodies. Abovementioned findings, accompanied by the MRI scans showing multiple infarcts in the hemispheres and basal ganglia, enabled to diagnose of primary APS with cortical and subcortical infarction, as a possible cause of CBS-like symptoms (Morris and Lees, 1999). Martino et al. (2006) described the second case-a 56-year-old woman with limb-kinetic apraxia and bradykinesia, whose brain MRI showed white matter changes, diffuse cerebral atrophy and several infarcts. Relatively similar medical case was described by Hashimoto et al., who presented a 53-year-old woman, who developed CBS due to APS secondary to SLE. Motor symptoms, which occurred in this patient were later attributed to the subcortical ischemic changes, cortical atrophy and several small infarctions in white matter; no basal ganglia infarcts were revealed (Hashimoto et al., 2018). One case of CBS associated with APS occurred without cerebral infarctions (Lee et al., 2014). It may be concluded that cerebrovascular lesions due to thrombosis are associated with CBS, while high levels of antiphospholipid antibodies, which damage vascular and attack the basal ganglia, contribute to development of CBS.

In addition, the literature reviews report the coexistence of CBS with Hashimoto encephalopathy. In the paper presented by Jain et al., a 70-year-old woman developed CBS symptoms in the course of Hashimoto encephalopathy (Table 1). An MRI scan showed cortical ribboning. She had a high titer of antithyroid peroxidase antibodies (Jain and Khan, 2017). Sheetal et al. (2016) also described the case of the patient with a high level of antibodies and CBS-like symptoms. These data suggest that high level of anti TPO antibodies, which damage vascular endothelium and lead to perivascular infiltrations in cerebral cortex, may contribute to the development of CBS (Stojanovich et al., 2013; Ricarte et al., 2018). Nevertheless, CBS related to Hashimoto’s encephalopathy was described only twice. It is possible that autoimmune processes, which lead to onset of vascular pathology and perivascular infiltrates, causes anoxia and brain edema, which further causes manifestations of CBS (Jain and Khan, 2017).

Vascular malformations have also been reported as a cause of CBS. Cavernous angioma described by Bihari et al. (2016) and dural arteriovenous fistula observed by Takase et al. (2017) were associated with neurological symptoms (Table 1). In patients, who developed symptoms of CBS associated with vascular malformations mechanism remains unclear (Bihari et al., 2016).

#### Molecular and Genetic Studies

Apart from the evidence acquired from the clinical practice it was observed that a specific endothelial growth factor may serve as a risk factor of CBS (Chahine et al., 2014). Jin et al. (2000) proved that VEGF may protect neurons from hypoxic or ischemic injury. The results of the study by Borroni, support a role for specific VEGF polymorphisms and haplotypes as risk factors for neurodegenerative diseases. Also, this study suggests the key role of genetic background in CBS pathogenesis proving that AGG (-2578C/A, -1190G/A, -1154G/A) haplotype carriers had a 6-fold increased risk to develop CBS than non-carriers. These data suggest that specific genetic variations of VEGF are a predisposing risk factor for CBS (Borroni et al., 2010).

## Discussion

### Clinical Perspective

According to our research, vascular CBS, despite being relatively uncommon, should be considered in the differential diagnosis in patients with possible diagnosis of CBD and other neurodegenerative diseases. There are several indicators, which can implicate different than neurodegenerative etiology of CBS, vascular one included. Patients with vascular CBS tend to present a rapid onset of the symptoms, comparing to primarily insidious clinical phenotype of CBD. Moreover, their neurological state may deteriorate in a precipitous manner, as subsequent vascular episodes occur (Engelen et al., 2011). The presence of the time gap between a stroke and the appearance of neurological symptoms should not be perceived as an argument against vascular basis of CBS, as additional silent cerebrovascular accidents, typical for high-risk patients, may collectively contribute to exacerbation of their health state (Koga et al., 2019). According to our review, there is no typical clinical phenotype for vascular CBS, as patients experience a wide range of symptoms, from motor abnormalities to higher cortical dysfunctions. Nevertheless, it can be observed that individuals from this group are more likely to suffer from hypertension, dyslipidemia and diabetes mellitus; also, they often have a history of ischemic incidents and vascular disease (Table 1). Additionally, vascular etiology of CBS should be taken into consideration when the clinical manifestation includes other than typical CBS symptoms, as their presence may arise from cerebrovascular disease localized in various brain regions simultaneously (Morris and Lees, 1999; Martino et al., 2006). In spite of the fact that vascular CBS results from often irreversible brain damage, it is possible for some patients to improve after certain medical procedures, which enhance cerebral blood flow (Kim et al., 2009). On the other hand, patients with vascular CBS show little to no therapeutic response to L-dopa treatment, similarly to patients with CBS (Marsili et al., 2016).

## Conclusion

Based on the cited studies, we concluded that the main risk factors of vascular CBS are ischemic. All various vascular pathologies described in this article lead to the conclusion that cerebral hypoperfusion can play a significant role in neuropathological changes, whose clinical manifestations can be various, CBS included. Nevertheless, it should be mentioned that the underlying disease may coexist with other pathologies, which do not necessarily influence its course or severity. Similarly, the number of lesions in the brain may not correlate with the severity of clinical manifestation. Many co-pathologies were described in CBD and PSP and pure syndromes in these neurodegenerative diseases are rare, but it is suspected that the rate of disease progression and course of main symptoms mostly depends on tau pathology (Jecmenica Lukic et al., 2020; Robinson et al., 2020). Coexistence of multiple possible causes of clinical symptoms hinders the diagnostic process and makes the evaluation of their significance troublesome. Another limitation of our study is the absence of post-mortem pathological examination in the majority of cited studies, which precludes assessing the real contribution of neurodegeneration in the clinical picture of presented patients. Nonetheless, our study has a chance to broaden the knowledge in a field of CBS pathogenesis and revise the way movement disorders are perceived. Presumably, the analysis of the holistic picture of coexisting symptoms, co-pathologies and genetic polymorphisms may improve the diagnostic process (Robinson et al., 2020). The importance of our paper derives from the fact that the prevalence of vascular incidents is high in the population, but rarely they are associated with CBS clinical phenotype. In addition to limited measures to confirm CBD diagnosis, which is commonly perceived as the main cause of CBS, it can lead to certain extent of misdiagnosis. We are positive that our research will encourage the Clinicians to consider vascular etiology in patients with CBS and consequently provide them with appropriate medical care. Moreover, as possible ways of vascular incidents’ prevention exist, our research, compelling attention to vascular background, will hopefully contribute to the reduction of the number of patients with CBS.

## Author Contributions

AD involved in the study design, data analysis, review of the literature, writing – original draft preparation, writing – review and editing, and final acceptance. KW, KS, and JP involved in the data analysis, review of the literature, writing – original draft preparation, and writing – review and editing. PA involved in the study design, writing – review and editing, supervision, and final acceptance. All authors contributed to the article and approved the submitted version.

## Conflict of Interest

The authors declare that the research was conducted in the absence of any commercial or financial relationships that could be construed as a potential conflict of interest.

## References

[B1] Abe Y. Kimura N. Goto M. Aso Y. Matsubara E. (2016). Brain perfusion in corticobasal syndrome with progressive aphasia. *Dement. Geriatr. Cogn. Dis. Extra.* 6 133–141. 10.1159/000443329 27195001 PMC4868931

[B2] Ali F. Josephs K. A. (2018). Corticobasal degeneration: key emerging issues. *J. Neurol.* 265 439–445. 10.1007/s00415-017-8644-3 29063240

[B3] Arendt T. Stieler J. T. Holzer M. (2016). Tau and tauopathies. *Brain Res. Bull.* 126 238–292. 27615390 10.1016/j.brainresbull.2016.08.018

[B4] Armstrong M. J. Litvan I. Lang A. E. Bak T. H. Bhatia K. P. Borroni B. (2013). Criteria for the diagnosis of corticobasal degeneration. *Neurology* 80 496–503. 23359374 10.1212/WNL.0b013e31827f0fd1 PMC3590050

[B5] Baborie A. Griffiths T. D. Jaros E. Mckeith I. G. Burn D. J. Richardson A. (2011). Pathological correlates of frontotemporal lobar degeneration in the elderly. *Acta Neuropathol.* 121 365–371. 20978901 10.1007/s00401-010-0765-z

[B6] Bihari J. Hornyák C. Szöke K. Vajda J. Bagó A. G. Várallyay G. (2016). Corticobasal syndrome due to superficial siderosis caused by thalamic cavernoma. *J. Neuropsychiatry Clin. Neurosci.* 28 e15–e16. 26844964 10.1176/appi.neuropsych.15080196

[B7] Boeve B. F. (2011). The multiple phenotypes of corticobasal syndrome and corticobasal degeneration: implications for further study. *J. Mol. Neurosci.* 45 350–353. 10.1007/s12031-011-9624-1 21853287

[B8] Borroni B. Del Bo R. Goldwurm S. Archetti S. Bonvicini C. Agosti C. (2010). VEGF haplotypes are associated with increased risk to progressive supranuclear palsy and corticobasal syndrome. *J. Alzheimers Dis.* 21 87–94. 10.3233/jad-2010-091615 20413880

[B9] Broce I. J. Tan C. H. Fan C. C. Jansen I. Savage J. E. Witoelar A. (2019). Dissecting the genetic relationship between cardiovascular risk factors and Alzheimer’s disease. *Acta Neuropathol.* 137 209–226. 30413934 10.1007/s00401-018-1928-6 PMC6358498

[B10] Carecchio M. Cantello R. Comi C. (2014). Revisiting the molecular mechanism of neurological manifestations in antiphospholipid syndrome: beyond vascular damage. *J. Immunol. Res.* 2014:239398. 10.1155/2014/239398 PMC3987798 24741580

[B11] Chahine L. M. Rebeiz T. Rebeiz J. J. Grossman M. Gross R. G. (2014). Corticobasal syndrome: five new things. *Neurol. Clin. Pract.* 4 304–312. 10.1212/cpj.0000000000000026 25279254 PMC4160446

[B12] Chand P. Grafman J. Dickson D. Ishizawa K. Litvan I. (2006). Alzheimer’s disease presenting as corticobasal syndrome. *Mov. Disord.* 21 2018–2022. 16977625 10.1002/mds.21055

[B13] De La Torre J. (2018). The vascular hypothesis of alzheimer’s disease: a key to preclinical prediction of dementia using neuroimaging. *J. Alzheimers Dis.* 63 35–52. 10.3233/jad-180004 29614675

[B14] De Reuck J. L. Deramecourt V. Cordonnier C. Leys D. Pasquier F. Maurage C. A. (2012). Cerebrovascular lesions in patients with frontotemporal lobar degeneration: a neuropathological study. *Neurodegener Dis.* 9 170–175. 10.1159/000335447 22377662

[B15] Di Stasio F. Suppa A. Marsili L. Upadhyay N. Asci F. Bologna M. (2019). Corticobasal syndrome: neuroimaging and neurophysiological advances. *Eur. J. Neurol.* 26 701–e752. 30720235 10.1111/ene.13928

[B16] Díaz-Ruiz C. Wang J. Ksiezak-Reding H. Ho L. Qian X. Humala N. (2009). Role of hypertension in aggravating abeta neuropathology of AD type and tau-mediated motor impairment. *Cardiovasc. Psychiatry Neurol.* 2009:107286. 10.1155/2009/107286 PMC2775676 19936102

[B17] Engelen M. Westhoff D. De Gans J. Nederkoorn P. J. (2011). A 64-year old man presenting with carotid artery occlusion and corticobasal syndrome: a case report. *J. Med. Case Rep.* 5:357. 10.1186/1752-1947-5-357 PMC3170346 21827686

[B18] Gibb W. R. Luthert P. J. Marsden C. D. (1989). Corticobasal degeneration. *Brain* 112(Pt 5) 1171–1192. 2478251 10.1093/brain/112.5.1171

[B19] Goedert M. Eisenberg D. S. Crowther R. A. (2017). Propagation of tau aggregates and neurodegeneration. *Annu. Rev. Neurosci.* 40 189–210. 10.1146/annurev-neuro-072116-031153 28772101

[B20] Grijalvo-Perez A. M. Litvan I. (2014). Corticobasal degeneration. *Semin. Neurol.* 34 160–173. 24963675 10.1055/s-0034-1381734

[B21] Hashimoto R. Ogawa T. Tagawa A. Kato H. (2018). Corticobasal syndrome associated with antiphospholipid syndrome secondary to systemic lupus erythematosus. *Case Rep. Neurol. Med.* 2018:5872638. 10.1155/2018/5872638 PMC5964410 29854505

[B22] Hossain A. K. Murata Y. Zhang L. Taura S. Saitoh Y. Mizusawa H. (2003). Brain perfusion SPECT in patients with corticobasal degeneration: analysis using statistical parametric mapping. *Mov. Disord.* 18 697–703. 10.1002/mds.10415 12784276

[B23] Hu W. T. Rippon G. W. Boeve B. F. Knopman D. S. Petersen R. C. Parisi J. E. (2009). Alzheimer’s disease and corticobasal degeneration presenting as corticobasal syndrome. *Mov. Disord.* 24 1375–1379. 19425061 10.1002/mds.22574

[B24] Irwin D. J. (2016). Tauopathies as clinicopathological entities. *Parkinsonism. Relat. Disord.* 22(Suppl. 1) S29–S33. 26382841 10.1016/j.parkreldis.2015.09.020 PMC4662611

[B25] Jain R. Khan I. (2017). Corticobasal syndrome like presentation of Hashimoto encephalopathy with cortical ribboning. *Indian J. Med. Special.* 8 209–212. 10.1016/j.injms.2017.08.003

[B26] Jecmenica Lukic M. Kurz C. Respondek G. Grau-Rivera O. Compta Y. Gelpi E. (2020). Copathology in progressive supranuclear palsy: does it matter? *Mov. Disord.* 35 984–993. 32125724 10.1002/mds.28011

[B27] Jin K. L. Mao X. O. Greenberg D. A. (2000). Vascular endothelial growth factor: direct neuroprotective effect in in vitro ischemia. *Proc. Natl. Acad. Sci. U.S.A.* 97 10242–10247. 10.1073/pnas.97.18.10242 10963684 PMC27841

[B28] Josephs K. A. Ishizawa T. Tsuboi Y. Cookson N. Dickson D. W. (2002). A clinicopathological study of vascular progressive supranuclear palsy: a multi-infarct disorder presenting as progressive supranuclear palsy. *Arch. Neurol.* 59 1597–1601. 10.1001/archneur.59.10.1597 12374498

[B29] Josephs K. A. Whitwell J. L. Boeve B. F. Knopman D. S. Petersen R. C. Hu W. T. (2010). Anatomical differences between CBS-corticobasal degeneration and CBS-Alzheimer’s disease. *Mov. Disord.* 25 1246–1252. 10.1002/mds.23062 20629131 PMC2921765

[B30] Khan S. Davies I. B. (2008). Hypoxia and Alzheimer disease. *CMAJ* 178 1687–1688. 10.1503/cmaj.1080038 18559809 PMC2413319

[B31] Kim Y. D. Kim J. S. Lee E. S. Yang D. W. Lee K. S. Kim Y. I. (2009). Progressive “vascular” corticobasal syndrome due to bilateral ischemic hemispheric lesions. *Intern. Med.* 48 1699–1702. 10.2169/internalmedicine.48.2415 19755778

[B32] Koga S. Roemer S. F. Kasanuki K. Dickson D. W. (2019). Cerebrovascular pathology presenting as corticobasal syndrome: an autopsy case series of “vascular CBS”. *Parkinsonism. Relat. Disord.* 68 79–84. 10.1016/j.parkreldis.2019.09.001 31621626 PMC7141792

[B33] Kovacs G. G. (2015). Invited review: neuropathology of tauopathies: principles and practice. *Neuropathol. Appl. Neurobiol.* 41 3–23. 10.1111/nan.12208 25495175

[B34] Kovacs G. G. (2017). Tauopathies. *Handb. Clin. Neurol.* 145 355–368. 28987182 10.1016/B978-0-12-802395-2.00025-0

[B35] Kreisler A. Mastain B. Tison F. Fénelon G. Destée A. (2007). [Multi-infarct disorder presenting as corticobasal degeneration (DCB): vascular pseudo-corticobasal degeneration?]. *Rev. Neurol. (Paris)* 163 1191–1199. 18355466 10.1016/S0035-3787(07)78403-5

[B36] Lee D. W. Eum S. W. Moon C. O. Ma H. I. Kim Y. J. (2014). Corticobasal syndrome associated with antiphospholipid syndrome without cerebral infarction. *Neurology* 82 730–731. 10.1212/wnl.0000000000000152 24443453

[B37] Liesz A. (2019). The vascular side of Alzheimer’s disease. *Science* 365 223–224. 31320524 10.1126/science.aay2720

[B38] Ling H. O’sullivan S. S. Holton J. L. Revesz T. Massey L. A. Williams D. R. (2010). Does corticobasal degeneration exist? *Clinicopathol. Eval. Brain* 133 2045–2057. 10.1093/brain/awq123 20584946

[B39] Marques A. Bourgois N. Vidal T. Ferrier A. Mathais S. Merlin C. (2018). Subacute corticobasal syndrome following internal carotid endarterectomy. *Rev. Neurol. (Paris)* 174 157–161. 10.1016/j.neurol.2017.06.017 29153271

[B40] Marsili L. Dickson D. W. Espay A. J. (2018). Globular glial tauopathy may be mistaken for corticobasal syndrome-pointers for the clinician. *Mov. Disord. Clin. Pract.* 5 439–441. 10.1002/mdc3.12634 30838299 PMC6336378

[B41] Marsili L. Suppa A. Berardelli A. Colosimo C. (2016). Therapeutic interventions in parkinsonism: corticobasal degeneration. *Parkinsonism. Relat. Disord.* 22(Suppl. 1) S96–S100. 26382843 10.1016/j.parkreldis.2015.09.023

[B42] Martino D. Chew N. K. Mir P. Edwards M. J. Quinn N. P. Bhatia K. P. (2006). Atypical movement disorders in antiphospholipid syndrome. *Mov. Disord.* 21 944–949. 10.1002/mds.20842 16538618

[B43] Mathew R. Bak T. H. Hodges J. R. (2011). Screening for cognitive dysfunction in corticobasal syndrome: utility of Addenbrooke’s cognitive examination. *Dement. Geriatr. Cogn. Disord.* 31 254–258. 10.1159/000327169 21474935

[B44] Miyaji Y. Koyama K. Kurokawa T. Mitomi M. Suzuki Y. Kuroiwa Y. (2013). Vascular corticobasal syndrome caused by unilateral internal carotid artery occlusion. *J. Stroke Cerebrovasc. Dis.* 22 1193–1195. 10.1016/j.jstrokecerebrovasdis.2012.07.005 22938697

[B45] Mokri B. Ahlskog J. E. Fulgham J. R. Matsumoto J. Y. (2004). Syndrome resembling PSP after surgical repair of ascending aorta dissection or aneurysm. *Neurology* 62 971–973. 10.1212/01.wnl.0000115170.40838.9b 15037703

[B46] Morris H. R. Lees A. J. (1999). Primary antiphospholipid syndrome presenting as a corticobasal degeneration syndrome. *Mov. Disord.* 14 530–532. 10.1002/1531-8257(199905)14:3<530::aid-mds1030>3.0.co;2-8 10348488

[B47] Mulroy E. Jaunmuktane Z. Balint B. Erro R. Latorre A. Bhatia K. P. (2020). Some new and unexpected tauopathies in movement disorders. *Mov. Disord. Clin. Pract.* 7 616–626. 10.1002/mdc3.12995 32775506 PMC7396854

[B48] Ogawa T. Fujii S. Kuya K. Kitao S. I. Shinohara Y. Ishibashi M. (2018). Role of neuroimaging on differentiation of parkinson’s disease and its related diseases. *Yonago Acta Med.* 61 145–155. 10.33160/yam.2018.09.001 30275744 PMC6158357

[B49] Okuda B. Tachibana H. Kawabata K. Takeda M. Sugita M. (2000). Cerebral blood flow in corticobasal degeneration and progressive supranuclear palsy. *Alzheimer Dis. Assoc. Disord.* 14 46–52. 10.1097/00002093-200001000-00006 10718204

[B50] Parmera J. B. Rodriguez R. D. Studart Neto A. Nitrini R. Brucki S. M. D. (2016). Corticobasal syndrome: a diagnostic conundrum. *Dement. Neuropsychol.* 10 267–275. 10.1590/s1980-5764-2016dn1004003 29213468 PMC5619264

[B51] Peters S. Eising E. G. Przuntek H. Müller T. (2001). Vascular parkinsonism: a case report and review of the literature. *J. Clin. Neurosci.* 8 268–271. 10.1054/jocn.2000.0807 11386806

[B52] Rabadia S. V. Litvan I. Juncos J. Bordelon Y. Riley D. E. Standaert D. (2019). Hypertension and progressive supranuclear palsy. *Parkinsonism. Relat. Disord.* 66 166–170. 31420308 10.1016/j.parkreldis.2019.07.036

[B53] Rebeiz J. J. Kolodny E. H. Richardson E. P. (1967). Corticodentatonigral degeneration with neuronal achromasia: a progressive disorder of late adult life. *Trans Am. Neurol. Assoc.* 92 23–26. 5634049

[B54] Rebeiz J. J. Kolodny E. H. Richardson E. P. (1968). Corticodentatonigral degeneration with neuronal achromasia. *Arch. Neurol.* 18 20–33. 10.1001/archneur.1968.00470310034003 5634369

[B55] Respondek G. Grimm M. J. Piot I. Arzberger T. Compta Y. Englund E. (2020). Validation of the movement disorder society criteria for the diagnosis of 4-repeat tauopathies. *Mov. Disord* 35 171–176. 31571273 10.1002/mds.27872 PMC7993399

[B56] Ricarte I. F. Dutra L. A. Abrantes F. F. Toso F. F. Barsottini O. G. P. Silva G. S. (2018). Neurologic manifestations of antiphospholipid syndrome. *Lupus* 27 1404–1414. 29768970 10.1177/0961203318776110

[B57] Robinson J. L. Yan N. Caswell C. Xie S. X. Suh E. Van Deerlin V. M. (2020). Primary tau pathology, not copathology, correlates with clinical symptoms in PSP and CBD. *J. Neuropathol. Exp. Neurol.* 79 296–304. 10.1093/jnen/nlz141 31999351 PMC7036659

[B58] Salminen A. Kauppinen A. Kaarniranta K. (2017). Hypoxia/ischemia activate processing of amyloid precursor protein: impact of vascular dysfunction in the pathogenesis of alzheimer’s disease. *J. Neurochem.* 140 536–549. 10.1111/jnc.13932 27987381

[B59] Saranza G. M. Whitwell J. L. Kovacs G. G. Lang A. E. (2019). Corticobasal degeneration. *Int. Rev. Neurobiol.* 149 87–136. 31779825 10.1016/bs.irn.2019.10.014

[B60] Sheetal S. K. Mathew R. Peethambaran B. (2016). Hashimoto’s encephalopathy as a treatable cause of corticobasal disease. *Ann. Indian Acad. Neurol.* 19 285–286. 10.4103/0972-2327.176859 27293355 PMC4888707

[B61] Stojanovich L. Kontic M. Smiljanic D. Djokovic A. Stamenkovic B. Marisavljevic D. (2013). Association between non-thrombotic neurological and cardiac manifestations in patients with antiphospholipid syndrome. *Clin. Exp. Rheumatol.* 31 756–760. 23899875

[B62] Takase K. I. Matsumoto S. Nishi H. Nakahara I. (2017). A case of superior sagittal sinus intracranial dural arteriovenous fistula mimicking corticobasal syndrome. *J. Neurol. Sci.* 376 91–92. 10.1016/j.jns.2017.03.004 28431635

[B63] Tan C. C. Zhang X. Y. Tan L. Yu J. T. (2018). Tauopathies: mechanisms and therapeutic strategies. *J. Alzheimers Dis.* 61 487–508. 10.3233/jad-170187 29278892

[B64] Thal D. R. Von Arnim C. A. Griffin W. S. Mrak R. E. Walker L. Attems J. (2015). Frontotemporal lobar degeneration FTLD-tau: preclinical lesions, vascular, and Alzheimer-related co-pathologies. *J. Neural. Transm. (Vienna)* 122 1007–1018. 10.1007/s00702-014-1360-6 25556950 PMC6655426

[B65] Tisel S. M. Ahlskog J. E. Duffy J. R. Matsumoto J. Y. Josephs K. A. (2020). PSP-like syndrome after aortic surgery in adults (Mokri syndrome). *Neurol. Clin. Pract.* 10 245–254. 10.1212/cpj.0000000000000708 32642326 PMC7292559

[B66] Toledo J. B. Arnold S. E. Raible K. Brettschneider J. Xie S. X. Grossman M. (2013). Contribution of cerebrovascular disease in autopsy confirmed neurodegenerative disease cases in the National Alzheimer’s Coordinating Centre. *Brain* 136 2697–2706. 10.1093/brain/awt188 23842566 PMC3858112

[B67] Vitt J. R. Hamedani A. G. Horn S. Gannon K. P. Price R. S. Greene M. (2020). Acquired hemicerebral atrophy secondary to chronic internal carotid steno-occlusive disease: a case series. *Neurohospitalist* 10 38–42. 10.1177/1941874419859762 31839863 PMC6900652

[B68] Winikates J. Jankovic J. (1994). Vascular progressive supranuclear palsy. *J. Neural. Transm. Suppl.* 42 189–201. 10.1007/978-3-7091-6641-3_15 7964687

[B69] Yamauchi H. Kagawa S. Kishibe Y. Takahashi M. Higashi T. (2017). Progressive cortical neuronal damage and extracranial-intracranial bypass surgery in patients with misery perfusion. *AJNR Am. J. Neuroradiol.* 38 935–941. 10.3174/ajnr.a5110 28255031 PMC7960377

[B70] Yamauchi H. Kudoh T. Kishibe Y. Iwasaki J. Kagawa S. (2007). Selective neuronal damage and chronic hemodynamic cerebral ischemia. *Ann. Neurol.* 61 454–465. 10.1002/ana.21104 17380523

[B71] Zhang L. Murata Y. Ishida R. Saitoh Y. Mizusawa H. Shibuya H. (2001). Differentiating between progressive supranuclear palsy and corticobasal degeneration by brain perfusion SPET. *Nucl. Med. Commun.* 22 767–772. 10.1097/00006231-200107000-00007 11453049

